# Metal chelation approach synergized with surfactant for spectrofluorimetric assay of moxifloxacin with method greenness evaluation

**DOI:** 10.1186/s13065-025-01580-5

**Published:** 2025-07-15

**Authors:** Sayed M. Derayea, Mohamed A. Hammad, Mahmoud A. Omar, Yasser F. Hassan

**Affiliations:** 1 Department of Analytical Chemistry, Faculty of Pharmacy, Minia University, Minia, 61519 Egypt; 2 Department of Pharmaceutical Analytical Chemistry, Faculty of Pharmacy, Minia National University, New Minia, Egypt; 3 Department of Pharmaceutical Analytical Chemistry, Faculty of Pharmacy, Sadat City University, Monufia, 32958 Egypt; 4 Pharmacognosy and Pharmaceutical Chemistry Department, College of Pharmacy, Taibah University, Al-Medinah Al-Mounawarah, Medina, 30078 Saudi Arabia; 5 Department of Pharmaceutical Analytical Chemistry, Faculty of Pharmacy, Al-Azhar University, Assiut branch, Assiut, 71524 Egypt

**Keywords:** Spectrofluorimetry, Moxifloxacin, Aluminum ions, Sodium Lauryl sulfate, Method greenness

## Abstract

This work deals with devising a quick and easy method for moxifloxacin assay using a sensitive spectrofluorimetric technique. The method benefited from the interaction of the aforementioned drug with the aluminum cation which form a stable chelate with a high fluorescence characteristic. Further improvement in the fluorescence was achieved upon the addition of sodium lauryl sulfate. The experimental parameters of the reaction were carefully investigated. The fluorescence intensity was measured at a wavelength of 478 nm after excitation at 365 nm. A linear relationship between the intensity of fluorescence and moxifloxacin concentration was in the range of 10–200 ng mL^− 1^. The developed method was highly sensitive since the lower limit of detection was 2.2 ng mL^− 1^. Application of the suggested spectrofluorimetric method included the analysis of commercial tablet dosage forms containing the cited drug. In addition, human plasma samples spiked with the drug were analyzed, and good recovery was obtained. Moreover, the environmental friendliness of the developed procedure was confirmed through employing AGREE and GAPI approaches.

## Introduction

The fluoroquinolone group is considered an important therapeutic agent that has effective anti-bacterial properties and is readily absorbed from the gastrointestinal tract; thus, the bioavailability of these drugs is high [1, 2]. This group of antibiotics prevents bacterial DNA replication and transcription by inhibiting two important bacterial enzymes, topoisomerase II and IV [3, 4]. As a fourth-generation fluoroquinolone, Moxifloxacin (MOX) shows promise in treating a range of nosocomial and community-acquired diseases. Additionally, it seems that this drug overcomes bacterial resistance to fluoroquinolones of the second and third generations [5].

Different analytical methods were previously published for the assay of MOX. These methods include: titrimetry [6, 7], spectrophotometry [8–13], spectrofluorimetry [14–19], HPLC [20–24], capillary electrophoresis [25–27], potentiometry [28] and voltammetry [29–31]. Chromatographic assay using HPLC requires time-consuming and troublesome sample treatment protocols for cleanup before the analysis. Additionally, the use of huge amounts of highly pure organic solvents increases both the cost of analysis and environmental pollution. Moreover, some of the published HPLC methods use mass detectors which are extremely expensive limiting their routine use [21, 23]. Alternatively, spectrofluorimetry represents a properly straightforward and relatively cheap analytical approach. The environmental safety of this technique could be greatly improved through the proper selection of reaction solvent and the utilized reagents. Most of the published spectrofluorimetric approaches for MOX assay were less sensitive than the proposed method [14–18]. In addition, one of these methods was nonselective being based on oxidation with Ce(IV) [18]. Meanwhile, two methods were indirect since both were based on the quenching effect of the drug on the fluorescence of quantum carbon dots [15, 16]. Although the method reported by Ibrahim N. et al., [19] is sensitive enough, it is not suitable for practice in most laboratories since the fluorescence measurements should be carried out at low temperature (7.5 °C).

In order to get the aforementioned goals, it was necessary to increase the intrinsic fluorescence of the medication under study using different approaches. The first was to impede the PET process of the piperidine moiety that quench the fluorescence of the quinolone fluorophore moiety. Then, restricting the free rotation of the molecule through chelate formation with aluminum ions. Lastly, the use of surfactant inhibits fluorescence quenching through its electrostatic interaction with MOX which prevent energy transfer from MOX to the surrounding media. By using the three approaches together, it was possible to enhance the fluorescence intensity of MOX by about 4 folds compared with its aqueous solution. In view of these approaches, a novel, highly sensitive, and selective spectrofluorimetric approach was designed. The suggested method was employed for MOX assay in tablet dosage forms and spiked human plasma. The developed method is advantageous compared to the previously published methods specially those based on HPLC. Water was the used solvent which is cheap, safe for both human and environment and readily available, in contrast to the organic solvents used in HPLC. The analysis time is short and the sample preparation is feasible beside it does not required lengthy or expensive tools. Furthermore, the utilized instrument is relatively cheap and easy to handle. Meanwhile HPLC methods require staff that has experience in handling the equipment, frequent parts replacement, and regular maintenance. Moreover, the present method offers excellent sensitivity without compromising specificity or precision.

## Experimental

### Apparatus

For measuring the fluorescence, SCINCO FluoroMate (FS-2, Korea) was utilized. A 150 W Xenon-arc lamp was the light source of the spectrometer, and the width of the slit was adjusted to 5 nm for both excitation and emission.

### Chemicals and reagents

All reagents and chemicals used were of analytically approved grade. Potassium aluminum sulfate (KAl(SO_4_)_2_·12H_2_O), sodium lauryl sulfate (SLS), citric acid, hydrochloric acid, phosphoric acid, and sodium hydroxide in addition to solvents including methanol, ethanol, isopropanol, acetonitrile, and dioxan were obtained from El-Nasr Chemicals Co. (Cairo, Egypt). An aqueous solution of 0.53 mM KAl(SO_4_)_2_·12H_2_O was prepared as a fresh solution in water, while SLS was prepared as 1.0% aqueous solution. Toerell - Stenhagen buffer solutions having different pH values (5.0–7.0) were prepared by mixing the appropriate quantity of 0.1 M solutions of phosphoric acid, citric acid, and sodium hydroxide. A suitable volume of 0.1 M solution of hydrochloric acid was added to adjust the pH. Plasma samples were generously obtained from Assiut Regional Blood Transfusion Center (Assiut City, Assiut, Egypt) and stored at -20 ºC. Delmoxa tablet (Delta Pharmaceutical Industries, Cairo, Egypt) labeled to have 400 mg MOX per tablet was purchased from a local Egyptian pharmacy.

### Standard drug solution Preparation

A stock solution of the drug that contains 100.0 µg mL^− 1^ of MOX was prepared through dissolving 10.0 mg of the drug in 100 mL of distilled water in a calibrated flask. Portions of the resulting stock solution were diluted with the same solvent to constitute working drug solutions that had concentrations in the recommended range. Both stock and working solutions were stable for at least one week when kept in the refrigerator at -4 ºC.

### Procedures

#### General assay procedure

In a series of 10 mL calibrated flasks, certain volumes of MOX working solution that give final concentrations ranging from 10 to 200 ng mL^− 1^ were added, followed by 2 mL of Toerell-Stenhagen buffer solution (pH 6.0). After that, the content was mixed with 1.0 and 0.8 mL of 0.53 mM Al(III) and 1% SLS solutions, respectively. Distilled water was added to complete the flask to its final capacity. The contents were mixed carefully and set aside for 10 min at room temperature. The intensity of fluorescence of the final solution was monitored at 478 nm after being excited at 365 nm. The same steps were followed to prepare the reagent blank, but without the addition of the standard drug solution. The intensities of fluorescence were plotted against the final drug concentrations in each solution to set up the calibration plot. Additionally, the obtained data were subjected to a linear equation curve’s fitting.

#### Procedure for estimating the reaction stoichiometry between Al(III) and MOX

Two master solutions that contain the same molar concentration (5.3 × 10^− 4^ M) of both Al(III) and MOX were constituted. Into 10 mL calibrated flasks, various volumes from the drug and the metal solutions were transferred in a complementary manner. After that, the remaining steps of the general assay procedure were performed. Blank experiments were carried out using the same steps, but no drug solutions were added. The obtained fluorescence intensity values of the chelate were normalized in relation to the readings of the blanks. The normalized fluorescence intensity values were plotted against the mole fractions of MOX to get the Job`s plot.

#### Application to tablet dosage form

Twenty units of the commercially available MOX tablet dosage forms were grinded, and an amount of the obtained fine powder equivalent to 10 mg of MOX was accurately weighed and located into a 100-mL volumetric flask. For extracting the drug, the mixture was sonicated with water for about 20 min. The content was totaled to the final volume with water. The solution was filtered, and a portion of the resulting filtrate was subjected to further dilution using water to prepare a sample solution having the recommended concentration. Finally, a specific portion of the diluted solution was analyzed with the general assay procedure.

#### Procedure for the analysis of spiked plasma of human

An aliquot of 1.0 mL of plasma was spiked with different standard drug solutions with varied concentrations. After mixing the content thoroughly, acetonitrile was added in an equal volume to precipitate the plasma protein. The contents were homogenized by vortex mixing for about one minute and centrifuged for ten minutes at 5000 rpm. An aliquot (1.0 mL) of the clear solution was subjected to analysis using the general assay procedure.

## Results and discussion

The quinolone nucleus is the actual fluorophore in MOX, which is attached to a diazabicyclononyl moiety at position 7. This moiety contains an electron-rich nitrogen atom in the secondary amine group distal to the quinolone. This makes the quinolone nucleus susceptible to an intramolecular PET process. When the amine group is in its free form in the alkaline medium, the lone pair electrons of the nitrogen atom could be transferred to the conjugated system of the quinolone. Therefore, upon deprotonation of the nitrogen atom of diazabicyclononyl in a relatively alkaline medium, the PET process can happen, causing a quenching of the fluorescence of the quinolone. However, when the pH changes from alkaline to acidic conditions, the diazabicyclononyl is protonated, and thus the PET process will be blocked. Consequently, the fluorescence of the quinolone nucleus is switched on. This behavior was reported with other quinolone fluorophores.

Metal chelation is one of the useful approaches that has been utilized for the determination and detection of several pollutants and biologically important analytes using either colorimetric or fluorimetric techniques [32]. MOX, as a quinolone, has strong ability to form strong chelates with metal ions because its chemical structure contains a carbonyl group in close proximity to the hydroxyl of the carboxylic group, both attached to the quinolone nucleus. Since the location for metal chelation is near the fluorophore (quinolone ring), metal chelation would be accompanied by an augmentation of the fluorescence. Al^3+^ was previously used as a fluorescence sensitizing agent for the determination of other fluoroquinolones [33, 34]. Therefore, in the present work, the fluorescence characteristics of MOX were enhanced through chelation with Al^3+^ ions.

Another strategy to enhance the intrinsic fluorescence of many fluorophores is the use of the surfactant sensitization approach. Usually, the surfactant sensitization approach is carried out at concentrations above critical micelle concentration (CMC) [35, 36]. However, in some cases, surfactant could also be utilized for the same purpose at concentrations lower than CMC [37–39]. Protonated MOX carries a positive charge and thus could interact with the surfactant anion carrying a negative charge through electrostatic forces [40]. This interaction reduces the movement of MOX molecule and thus increases its rigidity. Therefore, the energy transfer from the excited fluorophore to the surrounding solvent molecules is greatly reduced. Consequently, the addition of SLS could produce a further enhancement in the fluorescence of MOX.

These three strategies were employed collectively for designing a novel spectroflourimetric method for the MOX determination. The proposed methodology is advantageous compared to the previous methods in respect to its higher sensitivity, simpler, less time-consuming, and utilizing a cheap reagent.

### Fluorescence spectra

**Fig. 1:**
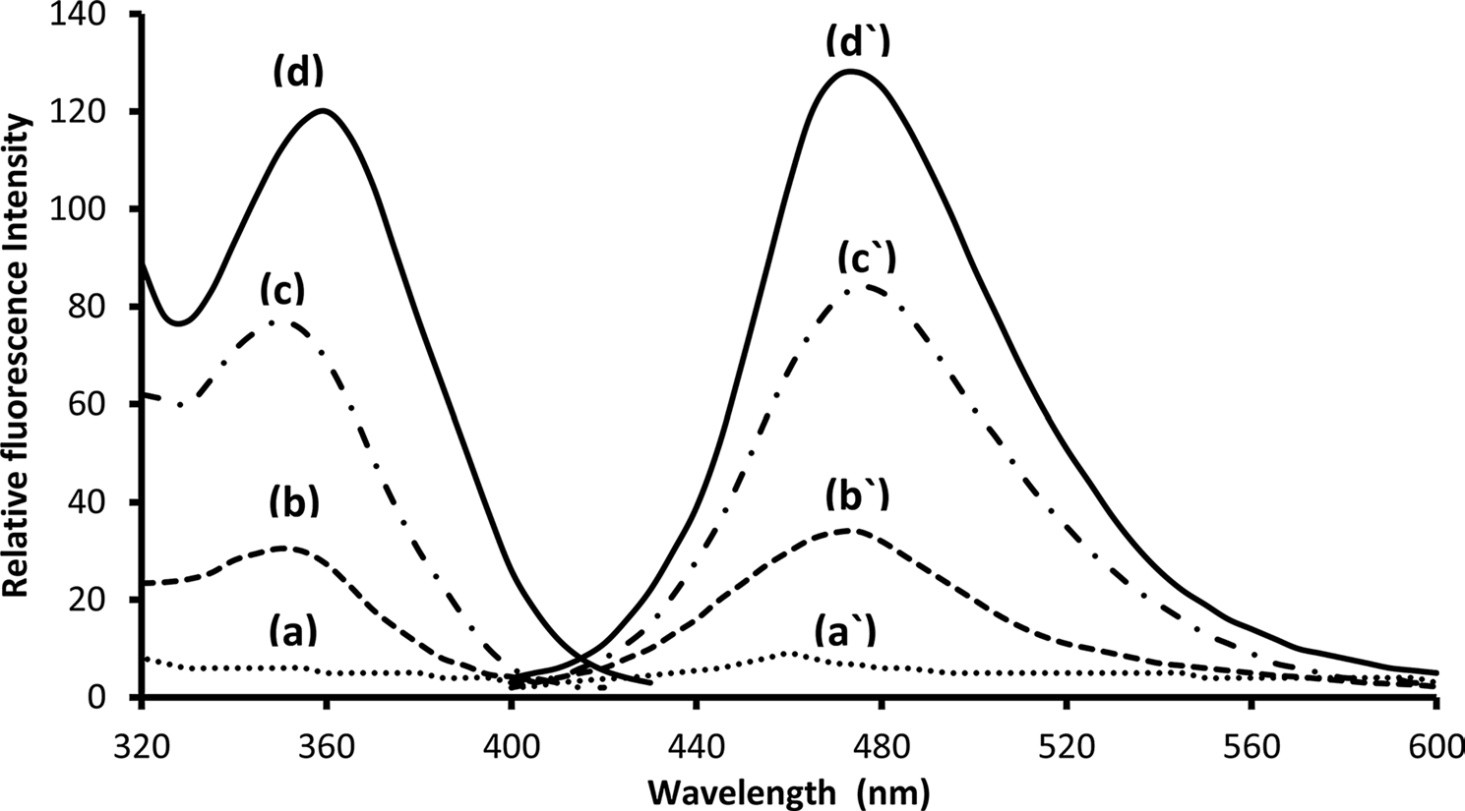
Excitation and emission spectra of Al(III)–SLS reagent (**a** and **a`**), 200 ng mL^− 1^ moxifloxacin in water, (**b** and **b`**), moxifloxacin– Al(III) (**c** and **c`**) and moxifloxacin - Al(III) - SLS system (**d** and **d`**)

In Fig. 1, the fluorescence spectra of Al(III)-SLS (curve a), MOX in aqueous solution (curve b), MOX-Al(III) (curve c) and MOX-Al(III)-SLS system (curve d) are presented. It could be seen that the fluorescence of MOX was greatly enhanced in the presence of Al(III) and SLS. The fluorescence intensity was located at a wavelength of 478 nm while the excitation wavelength was 365 nm.

### Optimization of the experimental variables

#### Effect of pH

**Fig. 2:**
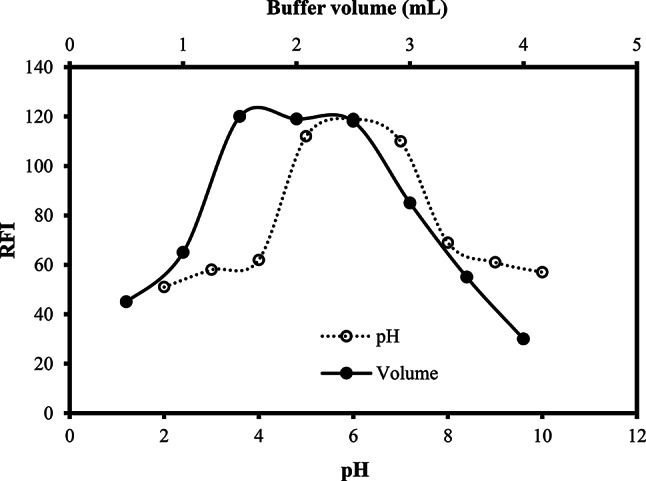
Effect of pH and volume of Teorell - Stenhagen buffer on the fluorescence intensity of reaction product of the MOX (200 ng/ml) with 0.53 mM Al(III) and 1% w/v SLS

The impact of the solution`s pH on the fluorescence of the reaction product was evaluated using Toerell & Stenhagen buffer solutions with different pH values (2.0–10.0). The highest fluorescence intensities were achieved at pH values ranging from 5.0 to 7.0. Using solutions having pH outside these values greatly diminishes the measured fluorescence. As a result, the use of a buffer system with pH 6.0 was recommended in the present work (Fig. 2). MOX is a hydrophilic compound that has two ionizable functionalities: carboxylic and secondary amine groups, and thus has two pka values, 6.25 (strong acidic) and 9.29 (strong basic) [5]. The low fluorescence intensity at high pH (>7.0) could be due to de-protonation of the secondary amino group in the diazabicyclononyl system. This would promote a photo-induced electron transfer process from the secondary amine lone pair to the quinolone nucleus. Thus the fluorescence would greatly diminish. While the neutral or slightly acidic medium (pH 5.0–7.0) ensures the protonation of the basic nitrogen atom of the diazabicyclononyl moiety and thus blocks the PET process. On the other hand, the low fluorescence at highly acidic pH (<5.0) may be attributed to the collisional quenching produced from H^+^ of the COO^−^ transported by the solvent molecules to the excited molecule. Additionally, the high fluorescence at pH values from 5.0 to 7.0 may partially come from the high stability of the formed aluminum chelate at this pH range.

#### Effect of volume of buffer solution

The influence of different volumes of the buffer system was inspected using 0.4–4.0 mL of Toerell - Stenhagen buffer (pH 6.0). The measured fluorescence attained the highest reading when the buffer volume was 1.5–2.5 mL. Therefore, the use of 2.0 mL of the buffer system was recommended (Fig. 2).

#### Effect of Al(III) solution concentration

**Fig. 3:**
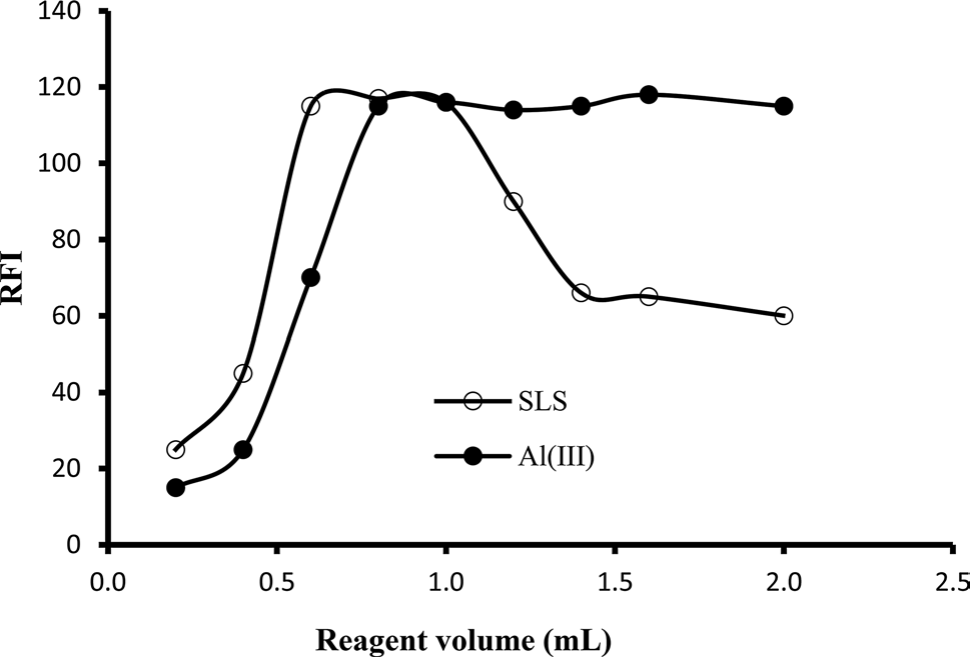
The influence of volume of 0.53 mM Al(III) and SLS (1%) reagents on the fluorescence intensity of reaction products of MOX (200 ng/ml) in presence of Teorell - Stenhagen buffer (pH 6.0)

The impact of Al(III) concentration on the intensity of fluorescence was tested using an altered volume of 0.53 mM Al(III) solution. It was noticed that rising the volume of Al(III) solution produced a related rise in the intensity of fluorescence up to 0.8 mL where maximum reading were observed. Further increase in Al(III) solution volume did not produce any clear change in the fluorescence (Fig. 3). Hence, 1.0 mL was the preferred volume of 0.53 mM Al(III) solution.

#### Effect of the type of surfactant

Different types of surfactant were examined through their incorporation in reaction medium. The investigation involved the use of anionic type (sodium lauryl sulfate, SLS), cationic type (cetyltrimethyl ammonium chloride) and non-ionic type (polyethylene glycol 6000). In addition, neutral macromolecules, including β-cyclodextrin and carboxymethyl cellulose were inspected. Fortunately, the studied reagents prompted a substantial rise in the intensity of fluorescence for MOX-Al(III) chelate (Table 1). Nevertheless, SLS made the maximum influence and therefore was nominated as a fluorescence booster in the study.

**Table 1:** The influence of diluting solvents, and the type of surfactants (0.2 M solution) on fluorescence intensity of the complex of 200 ng/ml MOX with 0.53 mM Al(III), in the presence of Toerell-Stenhagen buffer solution (pH 6.0)

Solvent	RFI	Surfactant ^a^	RFI
Water	112.7	Sodium lauryl sulfate (SLS)	118.7
Isopropanol	60.1	β-cyclodextrin	40.1
Methanol	71.9	cetyltrimethylammonium chloride	65.5
Ethanol	70.8	carboxymethyl cellulose	60.1
Acetonitrile	67.6	polyethylene glycol 6000	69.4
Dioxan	65.5	None	26.2

#### Effect of SLS volume

The surfactant plays a crucial role in the augmentation of the emission characters of MOX. Henceforth, different volumes (0.2–2.0 mL) of 1.0% SLS were utilized in performing the procedure of the general assay. A recognizable rise in the emission happened on rising the volume of surfactant solution (1% SLS), as seen in Fig. 3. A maximum emission was attained when 0.6–1.0 mL of the reagent were used. Consequently, the selected surfactant volume was 0.8 mL. Although this selected volume of surfactant correspond to a concentration of 2.77 × 10^− 3^ M, which was lower than the CMC of SLS (8 × 10^− 3^ M), it was able to boost efficiently the emission of the investigated drug. The surfactant can enhance the fluorescence of MOX through electrostatic interaction with the drug, limiting its free rotation and thus protecting its fluorescence from being quenched by the surrounding solvent molecules.

#### Effect of the diluting solvent, reaction time and temperature

The selection of the best solvent to dilute the produced metal chelate in the presence of the SLS included the use of several organic solvents in addition to water. The solvents used were ethanol, methanol, isopropanol, dioxin, and acetonitrile. The use of all the examined organic solvents resulted in low intensity of fluorescence. On the other hand, the use of water made the highest reading and thus was nominated as the recommended diluting solvent of the produced chelate (Table 1). Using water as a diluent medium gives the proposed approach many benefits, such as being eco-friendly, reduced price, and prevalent accessibility. These advantages make the suggested methodology practical and fulfill the green and sustainable chemistry rules.

**Fig. 4:**
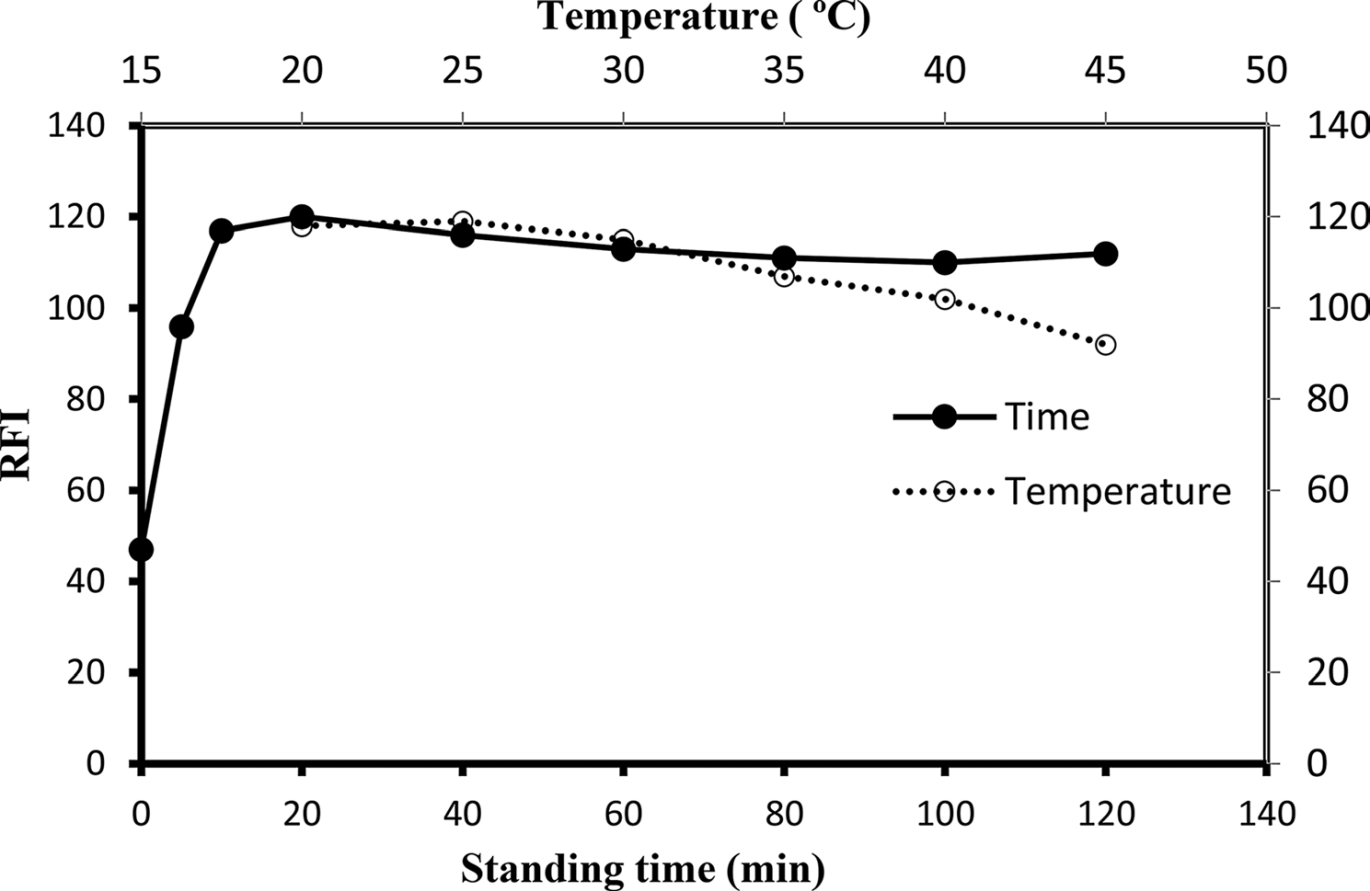
The influence of temperature and standing time on the stability of reaction products of MOX (200 ng/ml) with 0.53 mM Al(III) in presence of SLS (1%) and Teorell - Stenhagen buffer (pH 6.0)

The maximum fluorescence intensity was reached within 10 time after mixing the reactants and readings were not alter upon standing for at least 2 h (Fig. 4). Consequently, the emission measurements were carried out after 10 min of standing to ensure reproducible results. Furthermore, the reaction was carried out at different temperatures (20–45 ºC) as shown in Fig. 4. It was observed that higher temperature quench the fluorescence. Therefore, all subsequent works were performed at room temperature.

### Quantum yield of the formed complex

To determine the quantum yield of the formed complex between MOX and AL(III) in the presence of the surfactant, a comparative method was employed. A very diluted solution of fluorescein, which is well documented to have an estimated fluorescence quantum yield of 0.79, was taken as the reference standard. To estimate the quantum yield of the product, absorbance versus fluorescence intensity was plotted for fluorescein and the reaction product for various concentrations of the solutions. To avoid the inner filter effect, all solutions’ absorbance values were less than 0.1. The slopes ($\:{S}_{f}$ and S for fluorescein and formed complex, respectively) of the obtained linear plots were calculated and placed in the following quantum yield equation [41],

$${\varvec{Q}}={{\varvec{Q}}_{\varvec{f}}}\left[ {\frac{{\varvec{S}}}{{{{\varvec{S}}_{\varvec{f}}}}}} \right]{\varvec{x}}\left[ {\frac{{{\varvec{R}}{{\varvec{I}}^2}}}{{{\varvec{R}}{\varvec{I}}_{{\varvec{f}}}^{2}}}} \right]$$

Where, Q, S and RI are the quantum yield, slope of the plot, and solvent’s refractive index, respectively. The subscript “f” denotes fluorescein. Water was employed as the solvent for both fluorescein and the reaction medium. The quantum yield for the formed complex was calculated to be 0.27, which indicates the great fluorescence augmentation upon metal complexation and electrostatic interaction with surfactant.

### Investigating the reaction stoichiometry

**Fig. 5:**
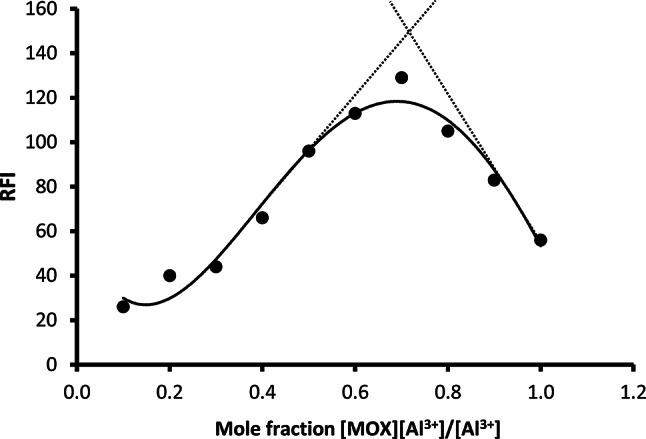
Job’s plot of equimolar solutions of MOX and Al(III) at the respective maxima

**Fig. 6:**
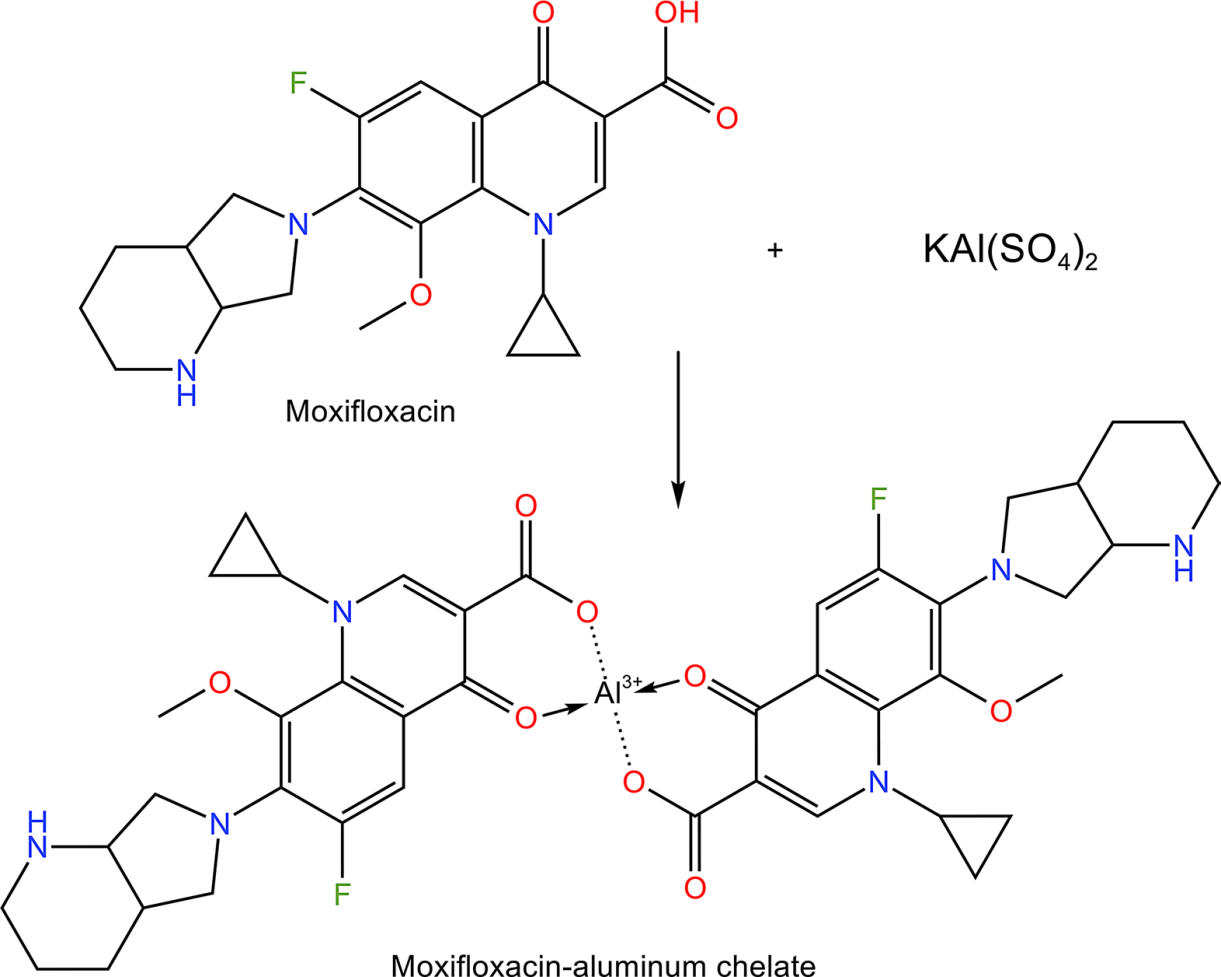
Chemical reaction for the chelate formation between MOX and Al(III) ions

The reaction stoichiometry between the drug and the metal ion was investigated through employing Job’s approach [42]. The procedure of the general assay was performed on solutions of both reactants having the same molar concentration (5.3 × 10^− 4^ M). Figure 5 shows that the intersection of the tangent lines to the Job curve is at a mole fraction of about 0.71, which indicates that the molar ratio between the aluminum ion and MOX in the resulting chelate is 1:2. This ratio is in agreement with the previously published work [43]. A suggested chemical reaction for the formation of the metal chelate between Al^3+^ and MOX is presented in Fig. 6.

### Causes of luminescence augmentation

Owing to the presence of rigid and planner quinolone moiety, MOX has an inherent fluorescence. In the present work, three different approaches were combined to collectively enhance the fluorescence of the investigated drug. The adjustment of solution pH to 6.0 ensures that the secondary amino group is in its protonated form, and thus the quenching effect that arises from the PET process is eliminated through PET blocking. Additionally, the chosen pH helps in attaining the suitable condition for the maximum chelate stability.

The fluorescence enhancement attained with Al^3+^ can be due to the chelation-induced fluorescence enhancement and the inhibition of an excited state intramolecular proton transfer (ESIPT) [44]. The chelation between MOX and Al^3+^ restricts the conformational flexibility at the excited state upon inhibition of C = O isomerization resulted in significant fluorescence enhancement. Chelating MOX with Al(III) ions also boosts the fluorescence of the drug through elevating the rigidity of the molecule and thus minimizes the loss of energy through vibration which is a non-radiative process [45]. Furthermore, the presence of a proton donor (such as -OH) in close proximity to a proton acceptor (-C = O) group generally facilitate the ESIPT process. The Al^3+^ binding to MOX switch off the ESIPT, which is characterized by a large Stock`s shift (by 113 nm) and remarkable fluorescence turned on (by ~ 2.5 fold).

Lastly, MOX could interact electrostatically with the surfactant which restrict the movement of the MOX-Al^3+^ chelate, and thus protect the formed chelate from energy loss to the neighbor solvent molecules [46]. Consequently, the usage of suitable pH, Al(III), and SLS produced synergistic roles for enhancing the intensity of fluorescence of MOX.

### DFT study

Gaussian 09 W program was used for DFT calculations using B3LYP/lanL2DZ level of theory. HOMO (highest occupied molecular orbital) and LUMO (lowest unoccupied molecular orbital) were obtained. The visualization of the investigated structure was done using GaussView visualization program.

**Fig. 7:**
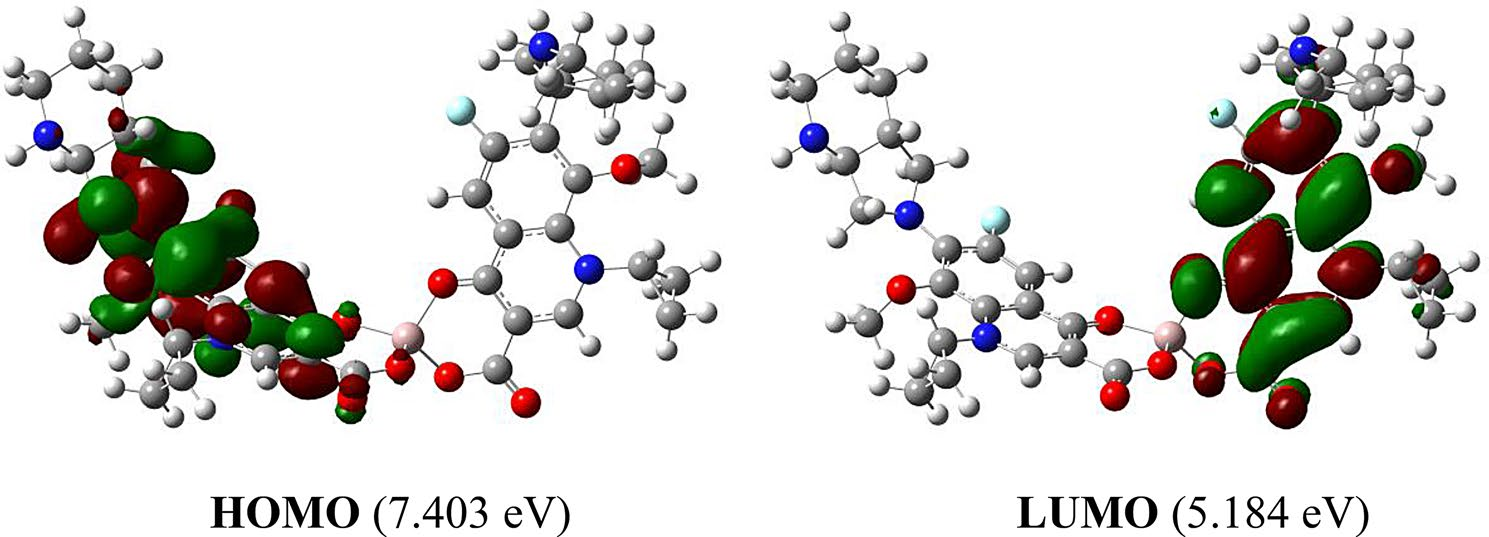
HOMO, LUMO and their energies of the formed complex between MOX and Al^3+^

LUMO is primarily an electron acceptor, while HOMO is an electron donor. Chemical stability in molecules is associated with the difference in energy between HOMO and LUMO. According to literature, those compounds that have a small energy gap are more chemically reactive, less kinetically stable, and softer in character, while compounds with the big energy gap exhibit the converse behavior. Figure 7 depicts the spatial orientations of HOMO, LUMO, and their energies, and the HOMO-LUMO gap. It was noted that the electron density of the HOMO is distributed over one MOX moiety of the complex, whereas the electron density of the LUMO is localized on the quinolone unit of the second MOX moiety. LUMO and HOMO energies are − 7.403 eV and − 5.184 eV, with a HOMO-LUMO gap of 2.219 eV. This gap is not high, so it is expected that the stability of the complex would be not so high.

### Method validation

The guidelines of ICH [47] were followed to analytically validate the developed spectrofluorimetric method.

#### Linearity, range, limit of detection and limit quantitaton

The procedure of general assay was applied for estimation of standard solutions having different concentrations of MOX. For setting up the calibration curve, the obtained intensity of fluorescence was related to MOX concentration. A relationship with high linearity (*r* = 0.9999) was established in MOX concentrations ranging from 10 to 200 ng mL^− 1^. The limits of detection (LOD) and quantitaton (LOQ) were estimated to check the sensitivity of the proposed methodology based on the intercept`s standard deviation (SD) and the calibration curve slope (b). The formulae utilized in the calculation were LOD = 3.3 SD/b and LOQ = 10 SD/b. The estimated values were 2.2 ng mL^− 1^ for LOD and 6.6 ng mL^− 1^ for LOQ. These values indicated that the proposed method is highly sensitive. Other statistical data are illustrated in Table 2.

**Table 2:** Analytical parameters for the determination of MOX with the proposed method

Parameter	Value
Excitation/emission wavelengths, (nm)	336/478
Linear range (ng/ml)	10–200
Slope	0.501
Standard deviation of slope	0.0028
Intercept	15.93
Standard deviation of intercept	0.328
Standard deviation of residuals SD × /y	0.4804
Coefficient of determination, r^2^	0.9989
Correlation coefficient, r	0.9995
Limit of quantitation (LOD^,^ ng/ml)	2.2
Limit of detection LOQ (ng/ml)	6.6

#### Accuracy and precision

The procedure of the general assay was performed within one day to analyze three standard solutions of MOX having varied concentrations to assess the accuracy and intraday precision of the developed method. For evaluating the inter-day precision, a standard solution of MOX having a specific concentration was analyzed in three different days. In all cases, the analysis was carried out three times, and the mean recovery percentage and relative standard deviation were calculated. It could be concluded from the values presented in Table 3 that the proposed method has high accuracy and suitable intra- and inter-day precision levels.

**Table 3:** Evaluation of accuracy, intraday and inter day precisions of the analytical procedure of the determination of MOX

Analysis	Taken ng/ml	Found ng/ml	% Recovery ± RSD ^b^	Er% ^c)^
	30	30.14	100.40 ± 0.54	0.40
Intra-day	50	49.99	99.97 ± 1.33	-0.03
	100	100.36	100.33 ± 1.53	0.33
	**Day**			
	**1**	50.16		
Inter-day (50 ng/ml)	**2**	50.19	100.35 ± 0.41	0.35
	**3**	49.82		

^*a*^ Average of three determinations, ^*b*^ Percentage relative standard deviation, ^*c*^ percentage relative error

#### Robustness

Minute changes in the experimental variable were introduced, and their effects on analytical performance were investigated to test the method`s robustness. The examined variables included solution pH, volumes of 0.53 mM aluminum ion and 1% SLS. The analysis was carried out three times and both % recovery and standard deviation were calculated. It was observed that the calculated values of the tested variables had no significant impact on the validity of the proposed method (Table 4).

**Table 4:** Robustness of the proposed method for determination of MOX (100 ng/mL)

Parameters	% Recovery ^a)^ ± SD ^b)^	
**No Variation**	101.13 ± 1.11	
**pH of buffer**		
6.2	100.55 ± 1.15	
5.8	99.98 ± 1.05	
**Al** ^**3+**^ **volume**		
0.8 mL	99.88 ± 1.15	
1.2 mL	98.78 ± 1.7	
**SLS volume**		
0.6 mL	100.55 ± 1.16	
1.0 mL	101.22 ± 1.99	

^*a)*^Average of three determinations, ^*b)*^ SD Standard deviation

#### Interference study

The selectivity of the developed method was assessed by checking how certain interferences could impact the result of the method (% recovery). Common co-formulated excipients were added to the drug standard solution. An aliquot containing 200.0 ng mL^− 1^ MOX (as a sample) was blended with 20 µg mL^− 1^ of each drug excipient individually (glucose, sucrose, fructose, maltose, and sodium tartrate) and the drug concentration in the resulting solution was determined using the developed method. The potential interference of other fluoroquinolones (pefloxacin, ofloxacin, levofloxacin and lomefloxacin) on the analysis of MOX was investigated. The fact that the % recovery values were close to 100% (as shown in Supplementary data, S2) guaranteed that there were no significant interferences from these excipients. However, all examined fluoroquinolones were severely affected the analysis with pefloxacin causing approximately five time increase in the percentage recovery. Thus, these drug should not present with MOX.

### Method`s applications

#### Assay of tablet dosage forms

The proposed approach was employed to analyze the available tablet dosage forms. The same tablet dosages were also analyzed using a reported method [14]. The obtained data from both methods were statistically compared to evaluate the accuracy and precision through calculating the values of Student`s t- and F-tests. The obtained data in Table 5 show that the calculated values of both tests did not exceed the reference values at the 95% confidence level. Consequently, the suggested method could be considered highly accurate and precise. Additionally, the obtained results verified the absence of any effect that could be encountered from the tablets` excipients.

**Table 5:** Application of the proposed method for the determination of the Moxifloxacin in Delmoxa 400 mg tablet dosage forms

Dosage form	% Recovery ± SD ^a^		t-test ^b^ value	F-test ^b^ value	$\:{\theta\:}_{L}$	$\:{\theta\:}_{U}$
	Proposed method	Reported method				
Delmoxa 400 mg tablet	99.57 ± 0.94	99.89 ± 1.53	0.30	2.55	0.9860	1.0207

^a^ The value is the mean of five measurements for proposed and three for reported methods, SD is standard deviation

^b^ Theoretical value at 95% confidence limit; F = 3.18 and t- =2.78

Bias in $\:{\theta\:}_{L}$ and $\:{\theta\:}_{U}$, based on recovery study of ± 2% is acceptable

Additionally, interval hypothesis was investigated, and the true bias implements on the recovery study was estimated using the following quadratic equation [48]:

$${\theta ^2}\left( {\bar {x}_{1}^{2} - S_{p}^{2}{t^2}/{n_1}} \right)2\theta {\bar {x}_1}{\bar {x}_2}+\left( {\bar {x}_{2}^{2} - ~S_{p}^{2}{t^2}/{n_2}} \right)=0$$

The lower ($\:{\theta\:}_{L}$) and upper ($\:{\theta\:}_{U}$) limits of the confidence interval were calculated according to the following formulae:

$${\theta _U}=~\frac{{ - b+\sqrt {{b^2} - 4ac} }}{{2a}}$$

$${\theta _L}=~\frac{{ - b - \sqrt {{b^2} - 4ac} }}{{2a}}$$

Where; $$a=~\left( {\bar {x}_{1}^{2} - S_{p}^{2}{t^2}/{n_1}} \right)$$

$$b=~2{\bar {x}_1}{\bar {x}_2}$$

$$c=~\left( {\bar {x}_{2}^{2} - S_{p}^{2}{t^2}/{n_2}} \right)$$

Where, $\:{\stackrel{-}{x}}_{1}^{2}\:$and $\:{\stackrel{-}{x}}_{2}^{2}\:$are values of the mean percentage recovery for the proposed and reported methods, respectively. $\:{n}_{1}$ and $\:{n}_{2}$ are the numbers of determinations. *Sp* is the pooled standard deviation and *t*- is the one-sided *t*-value at 95% confidence level. The calculated values of $\:{\theta\:}_{L}$ and $\:{\theta\:}_{U}$for the analysis are shown in Table 5 where these values were lower than ± 2 which is considered acceptable in the case of dosage forms analysis [35]. These results provided an additional evidence for the absence of any significant change in reliability of the suggested method.

#### Application to biological fluids

Human plasma samples were mixed with MOX standard solutions at three concentration levels. These concentrations lied in the ranges of the suggested spectofluorimetric method. Evaluation of accuracy was performed by recovery calculation of MOX at these concentration levels (three replicates of each concentration) for spiked plasma. After mixing MOX standard solution, plasma proteins were separated by the addition of acetonitrile and centrifugation, and then the resultant supernatants were subjected to analysis by the suggested spectrofluorimetric method in triplicate. The observed mean recovery percentage (± standard deviation) was 94.42 ± 2.89 (Table 6). These findings indicated the appropriateness of the recommended approach for the analysis of the specified drug in the spiked human plasma.

**Table 6:** Application of the proposed method for determination of MOX in spiked human plasma

Drug concentration (ng/mL)	%Recovery	Mean ± SD
30	94.15	
50	91.70	94.42 ± 2.89
100	97.45	

^*a)*^ The value is the average of three separate determinations

^*b)*^ SD is the standard deviation

### Evaluation of the method greenness

**Fig. 8:**
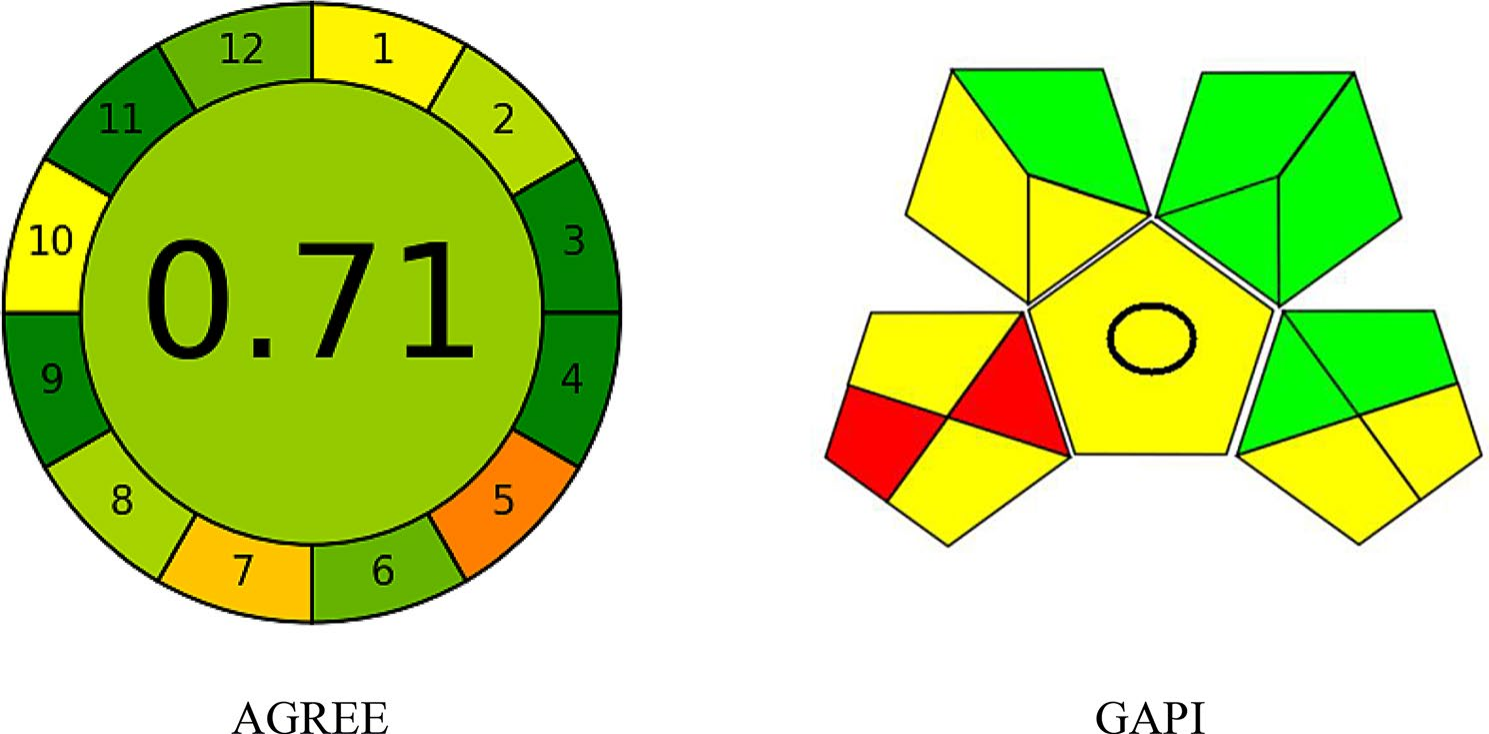
Evaluation of the greenness of the suggested spectrofluorometric approach using (**A**) GAPI (**B**) and AGREE metrics

AGREE [49] and GAPI [50–52] approaches were employed to assess the environmental friendliness of the developed procedure. The evaluation in GAPI includes the whole analytical process starting with samples collection and ending with the formal analysis. Every step was assessed and the result was illustrated in the form of pictogram having one of three different colors (green for ultimate green, yellow for neutral and red for non-green issues). As seen in Fig. 8, evaluation of the proposed methodology using GAPI gave 6 green areas, 7 yellow, and only 2 red, which reveal slight ecological influence of the tested method. In the evaluation using AGREE metric, the result is illustrated in the form of circular form look like a clock having a central part which indicates the overall evaluation result. From both its color and the number inside it, the total score of the evaluation could be obtained. While, 12 segments surround the central part, each of which represents the assessment of a certain parameter of analytical procedure. Each segment is graded in a scale from 0 with red color (non-green) to one with deep green color (ultimate green). The evaluation is straightforward and flexible, thanks to the free software. The methods’ greenness profiles using AGRee tool revealed that eight criteria out of twelve recommended them both as having the least hazardous and least harmful effect on the environment as shown in Fig. 8. In addition, the evaluated method was of high green level as raveled from the AGREE score of 0.71.

### Comparison with other spectrfluorimetric methods

Although there are different spectrofluorimetric methods that were published for analyzing MOX, many of them were less sensitive than the proposed method [14–18]. The method based on oxidation with Ce(IV) lacks selectivity because any reducing substance could greatly interfere with analysis [18]. On the other hand, two methods utilizing quantum carbon dots were indirect because the analytical signal is the quenching ability of MOX on the fluorescence of the reagent [15, 16]. Though the sensitivity of the method reported by Ibrahim N. et al., [19] is adequate, it has low practical suitability in most laboratories because a low temperature (7.5 °C) should be maintained during the fluorimetric measurements. Therefore, the merits of the suggested work relay on the high sensitivity combined with simple procedure and environmental safety for MOX assay in real samples. A comparison for the sensitivity of the suggested method with the previously reported fluorescence techniques is presented in Table 7.

**Table 7:** Comparison between the proposed method and the reported spectrofluorimetric methods for the determination of MOX

Technique/reagent	Linear range (ng mL ^− 1^)	LOD (ng mL^− 1^)	Ref.
Spectrofluorimetry/*Ce(IV)*	200–5000	16	[18]
Spectrofluorimetry/phosphate buffer (pH 8.3)	30–300	10	[14]
Spectrofluorimetry /Phosphate buffer (pH 9.7) at 7.4 °C	5–40	1.80	[19]
Synchronous spectrofluorimetry	50–500	14.9	[17]
Spectrofluorimetry /Carbon quantum dots	0.33–2.0 µM	2.59 nM	[16]
Spectrofluorimetry/Carbon quantum dots	0.025–15.0 µM	6.34 nM	[15]
Potentiometry with solid state electrode	0.44–439.9 µg.mL	240	[28]
Voltammetry/PtNPs electrode	10–1000 *µ*M	0.10 *µ*M	[29]
HPLC with fluorescence detection	2.5–500	34.84	[20]
LC-MS/MS	100–12,500	100	[21]
Capillary electrophoresis	50–5080	55	[27]
Colorimetry	1.89–40.0 µg/mL	644	[12]
Spectrofluorimetry/Al(III)/SDS	10–200	2.2	This work

## Conclusion

The proposed approach is rapid, economical, saves time and does not require a tedious sample extraction procedure before analysis. The use of water as reaction medium and diluent ensures the environmental safety and greenness of the proposed approach. Because it is highly sensitive, the developed methodology could be able to analyze bio-fluids with an acceptable recovery percentage. In the meantime, the absence of any interference from the excipients of tablet dosages and biological fluid components has confirmed the adequate selectivity of the proposed method. These features make the current method advantageous over many of the previously published spectrofluorimetric and spectrophotometric methods.

## Data Availability

No datasets were generated or analyzed during the current study.

## Abbreviations

AGREE: Analytical GREEnness (metric)
CMC: Critical micelle concentration
GAPI: Green analytical procedure index
HPLC: High performance liquid chromatography
ICH: International council on harmonization
LOD: Limits of detection
LOQ: Limit of quantitation
MOX: Moxifloxacin
PET: Photo induced electron transfer
RSD: Relative standard deviation
SD: Standard deviation
SLS: Sodium lauryl sulfate
